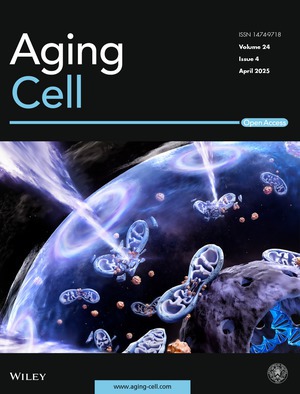# Featured Cover

**DOI:** 10.1111/acel.70072

**Published:** 2025-04-10

**Authors:** Zhidi Lin, Guangyu Xu, Xiao Lu, Hongli Wang, Feizhou Lu, Xinlei Xia, Jian Song, Jianyuan Jiang, Xiaosheng Ma, Fei Zou

## Abstract

The cover image is based on the article *Piezo1 exacerbates inflammation‐induced cartilaginous endplate degeneration by activating mitochondrial fission via the Ca2+/CaMKII/Drp1 axis* by Zhidi Lin et al., https://doi.org/10.1111/acel.14440.